# Soybean Rust Resistance and Yield Performance of Elite Soybean Genotypes Across Diverse Environments in Uganda

**DOI:** 10.1002/pei3.70125

**Published:** 2026-02-13

**Authors:** Tonny Obua, Julius Pyton Sserumaga, Godfree Chigeza, Alex Malaala, Mercy Namara, Bruno Awio, Solomon Okello, Phinehas Tukamuhabwa

**Affiliations:** ^1^ Department of Crop Science and Horticulture, School of Agricultural Sciences, College of Agricultural and Environmental Sciences Makerere University Kampala Uganda; ^2^ National Agricultural Research Organization National Livestock Resources Research Institute Kampala Uganda; ^3^ IITA Southern Africa Research and Administrative Hub (SARAH) Campus Chelston Zambia

**Keywords:** GEI, GGE biplots, grain yield, hundred‐seed weight, soybean rust resistance

## Abstract

Soybean ( *Glycine max* (L.) Merr.) is one of the important legumes globally, serving as an affordable and valuable protein source for humans and livestock. However, selecting the most suitable genotype across diverse environmental conditions remains a major challenge due to significant genotype‐by‐environment interactions (GEI). In addition, the quantitative inheritance of resistance to soybean rust and grain yield further complicates breeding efforts. This study aimed to assess the performance and stability of newly developed soybean genotypes regarding resistance to soybean rust and grain yield. Twenty‐two newly developed genotypes and two check varieties were evaluated using a randomized complete block design (RCBD) with three replications during five consecutive cropping seasons in six distinct locations in Uganda. GEI patterns were examined, and stable, high‐performing genotypes were found using genotype and genotype‐by‐environment (GGE) biplot analysis. The effects of genotype, environment, and genotype‐by‐environment interactions (GEI) on soybean rust resistance, hundred‐seed weight (HSW), and grain yield were all highly significant (*p* ≤ 0.01). The study revealed that genotypes 6N × SG‐P‐3‐2, 6N × SG‐P‐2, and 6N × SQ‐7 consistently performed better than all the other genotypes for soybean rust resistance, hundred seed weight, and grain yield across the six locations and five cropping seasons. Notably, these genotypes also demonstrated high stability for the three critical traits, making them strong candidates for varietal release. The results of this study provide valuable and new insights for soybean breeding programs in Uganda and the broader Sub‐Saharan Africa, offering a pathway for the development and release of rust‐resistant, high‐yielding soybean varieties adapted to varying agro‐ecological zones.

## 1 Introduction

Soybean ( *Glycine max* [L.] Merr.) ranks among the most significant legume crops globally, serving as an affordable protein source for both humans and animals, containing approximately 40% protein and 20% oil (Hartman et al. 2011). Soybean grain can be processed into various human food products and animal feeds, and its oil is the most widely consumed vegetable oil worldwide (Friedman and Brandon 2001; Singh et al. 2008). Globally, soybean contributes nearly 60% of total oilseed production and approximately 69% of protein meal consumed (SoyStat 2025). Overall, during the last two decades, soybean oil has recorded tremendous growth rates in terms of global consumption compared to other oils and fats (WWF 2014). Furthermore, a root symbiosis between soybean and rhizobia fixes atmospheric nitrogen into the soil, improving soil fertility (Hardarson and Atkins 2003). The nitrogen‐fixing capacity of soybean in the soil makes it an excellent crop for tropical Africa's sustainable agricultural system, which is characterized by low fertilizer use and infertile soils (Sanginga and Woomer 2009).

The fungus *Phakopsora pachyrhizi* is the primary cause of soybean rust disease, which is the world's largest challenge to soybean yield (Hossain et al. 2024; Murithi et al. 2016). Due to its rapid spread and the potential to cause significant yield losses, *P. pachyrhizi* is one of the most damaging foliar diseases affecting soybean (Hossain et al. 2024). Soybean rust causes rapid deterioration of leaf tissue, leading to early leaf senescence and drop, negatively affecting grain development. This disrupts grain development, resulting in smaller grains and ultimately reducing yield (Murithi et al. 2016). Enhancing grain yield and developing resistance to soybean rust are key goals of soybean breeding programs in Sub‐Saharan Africa (Tukamuhabwa et al. 2019; Tukamuhabwa and Maphosa 2011).

Several management strategies have been recommended to mitigate the negative impact of soybean rust disease (Godoy et al. 2016; Hossain et al. 2024). Some of these strategies, including fungicide use, cultural methods, and the adoption of resistant varieties, have been employed to manage soybean rust disease. While fungicide applications during the early vegetative stage of susceptible soybean plants can help reduce disease severity, they have generally shown limited success in improving yield (Faske et al. 2014; Sweets et al. 2008). This limited effectiveness is largely due to the timely application of fungicides—if they are applied after the disease is already established, yield outcomes tend to be inconsistent. Furthermore, the high costs and potential environmental hazards linked to fungicide use make them an impractical solution for disease management, particularly in resource‐limited regions such as Sub‐Saharan Africa. Cultural measures that reduce the disease's incidence include early planting, early‐maturing variety cultivation, timely irrigation, crop rotation, and the removal of alternate hosts (Pedersen 2005; Faske et al. 2014). However, the pathogen's broad host range and quick spread increase its survival, making cultural practices less effective (Hossain et al. 2024). Therefore, cultivation of resistant soybean varieties remains the most practical and sustainable strategy in the management of soybean rust disease in Sub‐Saharan Africa and other developing regions (Favoretto et al. 2025).

Several soybean breeding programs have conducted numerous multi‐environmental trials (METs) to evaluate soybean varieties and breeding lines for agronomic traits, including yield, seed composition, and resistance to pests and diseases. In Uganda, Makerere University Centre for Soybean Improvement and Development (MAKCSID) has selected multiple elite rust‐resistant soybean genotypes. Over the past decade, about ten METs have been conducted across Uganda to assess the adaptability of different elite lines in the major soybean production areas (Mukuze 2019; Obua et al. 2021; Tukamuhabwa et al. 2012). However, these studies have reported inconsistent findings regarding yield stability and the identification of optimal testing locations. These discrepancies can be attributed to the use of different genotype sets across trials and the increasing variability in climate conditions. The complexity is further compounded by the quantitative nature of soybean grain yield, which is highly influenced by genotype‐by‐environment interactions (GEI). This challenge is particularly pronounced when evaluations are conducted across diverse environments (Rani et al. 2023; Bianchi et al. 2020). GEI refers to the inability of genotypes to maintain consistent relative performance across varying environments (Baker 1988). Substantial GEI variation compromises the accuracy of soybean grain yield predictions and weakens the correlation between genotypic and phenotypic values, thereby limiting selection efficiency and genetic gain (Abebe et al. 2024).

In the presence of GEI, breeders can employ stability analysis to identify varieties that combine high yield with consistent performance across environments. Despite its widespread use, stability analysis often reduces the complex nature of GEI to simplified statistical indices, which may not fully capture the biological and agronomic relevance of genotype performance across diverse environments. Moreover, different stability models frequently produce inconsistent results, leading to varied interpretations and potential challenges in genotype selection (Annicchiarico 2002). Multi‐trait breeding tools, including selection indices, marker‐assisted selection (MAS), and quantitative trait loci (QTL) mapping, offer a valuable complement to stability analysis by facilitating simultaneous selection for multiple traits under varying environmental conditions. To analyze MET data, several statistical techniques have been developed. One of the most widely used techniques by plant breeders is the Genotype and Genotype‐by‐Environment Interaction (GGE) biplot analysis. GGE simultaneously displays genotype main effects (G) and genotype‐by‐environment interactions (GEI), and it is a robust technique for visual display of several queries resulting from MET data (Yan et al. 2007; Yan and Kang 2003). The GGE biplot approach has been widely applied to assess genotype‐by‐environment interactions in soybean.

Numerous studies have documented how soybean rust disease directly reduces grain yield (Gini‐Álvarez et al. 2025; Reis et al. 2019). Soybean rust reduces grain yield by causing leaf defoliation, reducing the production of assimilates (Reis et al. 2019). Moreover, the quantitative inheritance of both traits, as reported in previous studies, presents a significant challenge to improving them simultaneously in soybean. However, tolerance of soybean genotypes towards the soybean rust pathogen has been reported in previous studies. This implies that a soybean genotype can be attacked by the soybean rust pathogen yet still achieve a high grain yield. MAKCSID has identified and selected several elite rust‐resistant and high‐yielding soybean lines in the last five years. Therefore, this study aimed to assess the soybean rust resistance and yield stability of newly developed rust‐resistant soybean genotypes evaluated across six distinct locations in Uganda.

## 2 Materials and Methods

### 2.1 Experimental Materials

The soybean genotypes comprised 22 advanced breeding lines developed by the Makerere University Centre for Soybean Improvement and Development (MAKCSID), along with two check varieties, Maksoy 3N and Maksoy 6N (Table S1). Both check varieties are officially released in Uganda and are farmer‐preferred due to their large seed size, high grain yield, and rust resistance.

### 2.2 Description of the Locations Used in the Study

The trial was carried out across six locations, representing the key soybean‐growing regions of Uganda (Table 1). While Abi is in the North‐Western Farmlands Wooded Savanna, Bulindi is in the Western Grasslands, Ngetta is in the North‐Western Savannah Grasslands, and Mubuku is in the Western Medium High Farmlands, Kabanyolo and Nakabango are in the Lake Victoria Crescent. These locations exhibit varying climatic conditions that impact the severity of soybean rust disease and grain yield (Table 1). For example, the Mubuku irrigation scheme was selected to assess the adaptability of the soybean genotypes under irrigation conditions.

**TABLE 1:** Coordinates, altitude, annual temperature and annual rainfall for the six locations used to evaluate the 24 soybean genotypes for yield and agronomic traits in Uganda.

Location	Coordinates	Region	Altitude (masl)	Mean annual temperature (°C)	Mean annual rainfall (mm)	Soil type
Abi	3^o^04′N/30^o^56′ E	West Nile	1214	22.9	1404	Light sandy loam
Bulindi	1^o^41′N/31^o^42′ E	Mid‐West	1122	22.9	1355	Sandy loam
Kabanyolo	0^o^28′N/32^o^36′ E	Central	1180	21.4	1234	Sandy clay loam
Mubuku	0^o^13′N/30^o^08′ E	Western	1007	27.8	750	Sandy loam
Nakabango	0^o^29′N/33°14′ E	Eastern	1210	22.8	1400	Nitisol
Ngetta	2°17′N/32°56′ E	Northern	1103	24.7	1200	Sandy loam

Abbreviation: masl = meters above sea level.

*Source:* Meteorological station data at the study locations.

### 2.3 Experimental Design and Management of Experiment

To evaluate the performance of soybean genotypes under different field conditions, the experiment was laid out in a randomized complete block design (RCBD) with three replications. Each genotype was planted in three rows, each 5 m long, spaced 60 cm apart, with 5 cm between plants within each row. The multi‐environmental trials were conducted for five consecutive growing seasons: the second of 2022 (2022B), the first season of 2023 (2023A), the second season of 2023 (2023B), the first season of 2024 (2024A) and the second season of 2024 (2024B). The trial was kept free of weeds, with weeding carried out three times per season. No agrochemicals were applied for pest control, and standard management practices were followed consistently across all trial locations.

### 2.4 Data Collection

The trial was assessed for soybean rust resistance using a scale of 1–5 adopted from Favoretto et al. (2025), where 1 = highly resistant, 2 = resistant, 3 = moderately resistant, 4 = susceptible, and 5 = highly susceptible. Soybean rust resistance was evaluated between 70 and 75 days after planting, corresponding to the typical onset of disease pressure. The evaluation was conducted under natural soybean rust infestation and repeated each season throughout the experimental period to ensure consistent and reliable phenotypic assessment of the genotypes. At maturity, plants from all three rows of each genotype were harvested, manually threshed, and sun‐dried to a uniform moisture content of 10%. Subsequently, the hundred‐seed weight (HSW) and grain yield (tons ha^−1^) were assessed using OHAUS Scout SJX1502N/E portable weighing balance.

### 2.5 Data Analysis

Analysis of variance (ANOVA) for soybean rust resistance scores, hundred seed weight, and grain yield was conducted separately for each location and combined across locations using the *agricolae* package in R (R Teams 2025). The model treated genotypes as fixed effects, while replications and blocks within replications were considered random effects. For the combined analysis, the total variance was partitioned into appropriate sources to evaluate differences among genotypes and to assess the presence of genotype‐by‐environment interaction (GEI). Adjusted means for soybean rust resistance, hundred seed weight and grain yield obtained from ANOVA were subjected to GGE biplot analysis to partition the GEI for each trait and evaluate genotype performance stability across the different environments (Yan and Kang 2003; Yan and Rajcan 2002; Zhe et al. 2010). Soybean rust resistance data were transformed using a simple linear reversal method (*Max–x*) to ensure higher values reflect greater desirability (Derringer and Suich 1980; Harrington 1965). This method preserves the relative spacing and ranking among observations while reversing the scale.

## 3 Results

### 3.1 Soybean Rust Resistance Scores

#### 3.1.1 Performance of Genotypes Across Locations

The genotype, location, season and genotype × location interaction for soybean rust resistance were highly significant (*p* ≤ 0.001) as presented in Table 2. The soybean rust scores among the studied genotypes were generally low due to their high resistance to the rust disease in the field. Genotypes 6N × SQ‐7, 6N × SG‐P‐3‐2 and 6N × SG‐P‐2 are the best in soybean rust resistance as they recorded the lowest mean soybean rust scores of 1.6, 1.7 and 1.8, respectively (Table 3). On the other hand, genotypes Duiker × 3N‐4 and Namsoy 4M × 4N are the most susceptible, with mean soybean rust scores of 2.9 and 2.8, respectively (Table 3). Kabanyolo had the highest soybean rust resistance score of 3.1, followed by Mubuku (2.7) and Nakabango (2.5), and the lowest was recorded at Abi (1.0).

**TABLE 2:** Analysis of variance of soybean rust resistance scores of 24 soybean genotypes tested across six locations, for five cropping seasons in Uganda.

SOV	df	Sum sq	Mean sq	*F* value	Pr (> *F*)
Genotype	23	101.5	4.41	15.626	< 2e−16
Location	6	381.9	63.64	225.278	< 2e−16
Rep	2	0.6	0.3	1.068	0.344
Season	4	9.7	2.43	8.613	8.75e−07
Genotype × Location	134	89.6	0.67	2.367	7.27e−13
Genotype × Season	92	22	0.24	0.846	0.841
Location × Season	6	175.3	29.21	103.406	< 2e−16
Genotype × Location × Season	128	32.1	0.25	0.887	0.798
Residuals	682	192.7	0.28		

**TABLE 3:** Soybean rust resistance scores of 24 soybean genotypes tested across six locations for five cropping seasons in Uganda.

Genotype	Abi	Bulindi	Kabanyolo	Mubuku	Nakabango	Ngetta	Mean
6N × SQ‐7	1.0	1.5	1.9	1.6	1.8	1.3	1.6
6N × SG‐P‐3‐2	1.0	1.3	1.8	1.8	2.3	1.6	1.7
6N × SG‐P‐2	1.0	1.7	2.1	1.7	2.2	1.4	1.8
NG 14.1 × NII	1.0	1.7	3.1	2.2	2.3	1.7	2.1
6N × SL‐1‐2	1.0	1.5	2.9	2.4	2.2	1.9	2.1
Maksoy 6N	1.0	1.7	2.8	2.2	2.2	2.1	2.2
8.11 × 2N‐3	1.0	2.0	3.2	2.3	2.2	1.7	2.2
Wonder × 4M	1.0	1.7	3.5	1.9	2.8	1.6	2.2
Nam II × GC 17.3	1.0	2.3	2.9	2.3	2.4	1.8	2.3
Namsoy 4M × 2N	1.0	1.7	3.2	2.8	2.2	1.7	2.3
Maksoy 4N‐58‐1	1.0	1.8	3.3	2.9	2.5	1.6	2.4
Maksoy 6N × SL‐1	1.0	2.0	2.9	3.1	2.3	1.8	2.4
6N × SL	1.0	1.5	3.1	2.8	2.6	2.2	2.4
12.4 × NII‐1	1.0	1.8	3.2	3.1	2.7	1.5	2.4
UG5 × 4N‐1	1.0	1.8	3.3	3.1	2.4	1.9	2.4
UG5 × RPP5	1.0	1.8	3.6	3.2	2.4	1.5	2.5
8.11 × 2N	1.0	2.3	3.1	3.0	2.6	1.9	2.5
UG5 × 4M	1.0	2.3	3.4	2.8	2.6	1.8	2.5
Namsoy 4M × 14.1‐24	1.0	1.5	3.1	2.8	2.6	2.7	2.5
Maksoy 2N × 5N‐1	1.0	2.5	3.4	3.2	2.5	1.8	2.6
Maksoy 3N	1.0	1.5	3.5	3.4	2.6	2.1	2.6
6N × 2N‐2‐4‐1	1.0	1.8	3.6	3.6	2.6	2.1	2.7
Namsoy 4M × 4N	1.0	2.0	3.5	3.8	2.8	2.1	2.8
Duiker × 3N‐4	1.5	2.2	3.7	3.4	3.4	2.2	2.9
Mean	1.0	1.8	3.1	2.7	2.5	1.8	2.3

#### 3.1.2 Stability Using GGE Biplot Analysis

**FIGURE 1:**
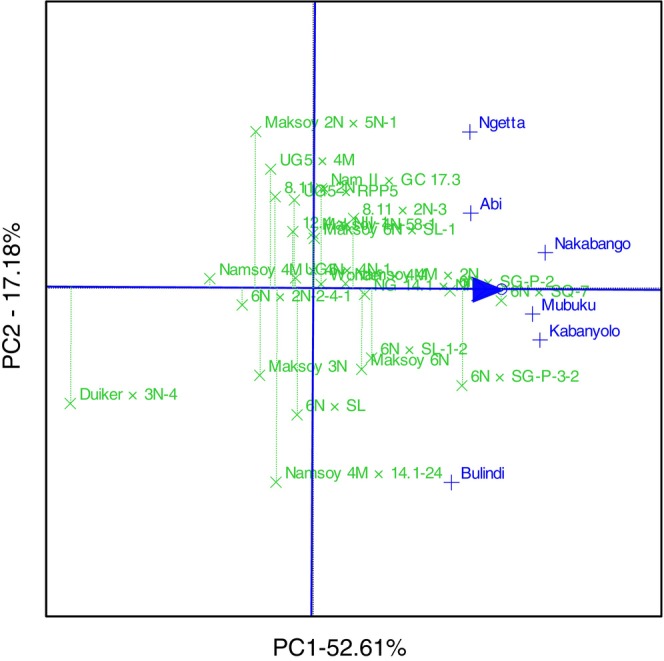
Performance and stability for rust resistance of 24 soybean genotypes tested across six locations for five cropping seasons in Uganda.

**FIGURE 2:**
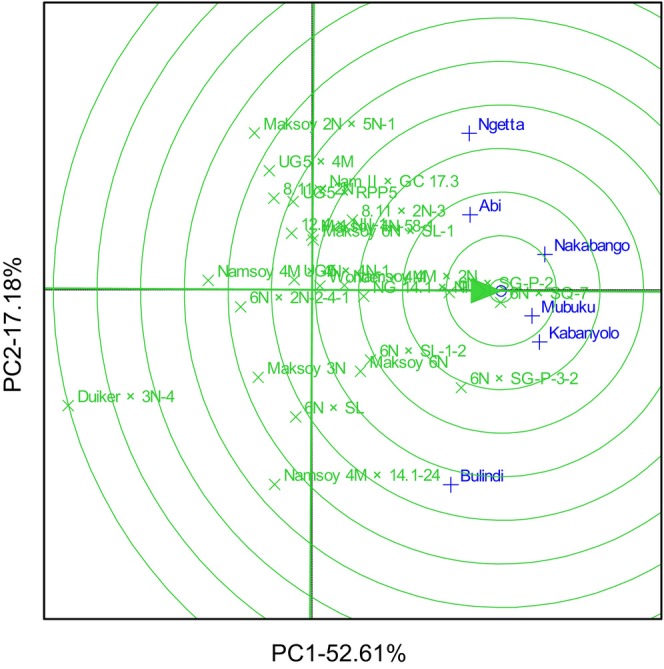
Ideal genotype analysis for rust resistance of 24 soybean genotypes tested across six locations for five cropping seasons in Uganda.

**FIGURE 3:**
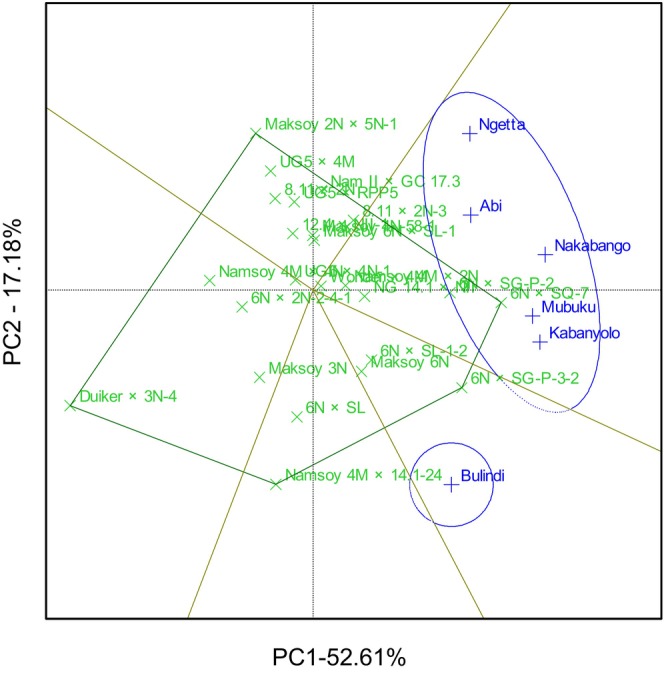
Mega environment analysis for rust resistance of 24 soybean genotypes tested across six locations for five cropping seasons in Uganda.

The GGE biplot explained 69.78% total GEI variation for rust resistance, identifying 6N × SG‐P‐3‐2 and 6N × SQ‐7 as the most resistant and stable genotypes. The biplot also identified Duiker × 3N‐4 as the most rust‐susceptible genotype (Figure 1). The GGE biplot also illustrated that 6N × SQ‐7 was the most ideal genotype, implying it had high and stable rust resistance in all six locations (Figure 2). The GGE biplot grouped the six locations into two major mega‐environments. The first mega‐environment included Abi, Ngetta, Nakabango, Mubuku, Kabanyolo, and 6N × SQ‐7, the vertex cultivar. The second mega‐environment included Bulindi with 6N × SG‐P‐3‐2 as the vertex cultivar (Figure 3).

### 3.2 Hundred Seed Weight (HSW)

#### 3.2.1 Performance of Genotypes Across Locations

The genotype, location, season and genotype × location interaction for hundred seed weight were highly significant (*p* ≤ 0.001) as presented in Table 4. Genotypes 6N × SG‐P‐3‐2 and 6N × SQ‐7 had the highest mean hundred‐seed weight of 20 g, followed by 6N × SG‐P‐2 with a mean HSW of 19 g. On the other hand, Namsoy 4M × 4N registered the lowest mean weight of 14 g (Table 5). Abi, Bulindi and Mubuku all recorded the highest mean HSW of 17 g while Nakabango recorded the lowest mean HSW of 15 g (Table 5).

**TABLE 4:** Analysis of variance of hundred seed weight of 24 soybean genotypes tested across six locations for five cropping seasons in Uganda.

SOV	df	Sum sq	Mean sq	*F* value	Pr (> *F*)
Genotype	23	2358	102.5	34.165	< 2e−16
Location	6	830	138.3	46.082	< 2e−16
Rep	2	61	30.5	10.155	4.51e−05
Season	3	3711	1237.1	412.225	< 2e−16
Genotype × Location	134	634	4.7	1.577	0.000154
Genotype × Season	69	248	3.6	1.198	0.139607
Location × Season	7	1085	155	51.639	< 2e−16
Genotype × Location × Season	151	472	3.1	1.041	0.366196
Residuals	677	2032	3		

**TABLE 5:** Hundred seed weight of 24 soybean genotypes tested across six locations for five cropping seasons in Uganda.

Genotype	Abi	Bulindi	Kabanyolo	Mubuku	Nakabango	Ngetta	Mean
6N × SG‐P‐3‐2	21	20	21	20	19	19	20
6N × SQ‐7	20	19	21	20	19	19	20
6N × SG‐P‐2	19	20	21	19	18	20	19
Namsoy 4M × 14.1‐24	18	20	18	18	16	17	18
Namsoy 4M × 2N	19	17	18	17	15	16	17
Maksoy 3N	19	16	17	17	16	17	17
6N × 2N‐2‐4‐1	19	17	16	17	14	18	17
Maksoy 2N × 5N‐1	17	19	17	16	16	17	17
NG 14.1 × NII	18	17	17	17	16	17	17
Nam II × GC 17.3	17	17	17	16	15	16	17
Maksoy 6N × SL‐1	18	16	15	17	16	16	17
6N × SL‐1‐2	18	16	16	17	16	16	16
Maksoy 6N	17	17	15	17	15	16	16
UG5 × RPP5	17	19	15	17	13	16	16
8.11 × 2N‐3	17	16	15	16	15	16	16
Maksoy 4N‐58‐1	17	16	16	16	16	16	16
6N × SL	17	16	15	17	14	16	16
UG5 × 4N‐1	18	17	15	16	13	15	16
Wonder × 4M	17	16	15	16	13	16	16
8.11 × 2N	16	14	15	17	15	16	16
12.4 × NII‐1	16	16	16	16	14	15	15
UG5 × 4M	16	17	15	16	14	14	15
Duiker × 3N‐4	14	15	13	14	12	15	14
Namsoy 4M × 4N	14	14	13	14	13	14	14
Mean	17	17	16	17	15	16	17

#### 3.2.2 Stability Using GGE Biplot Analysis

**FIGURE 4:**
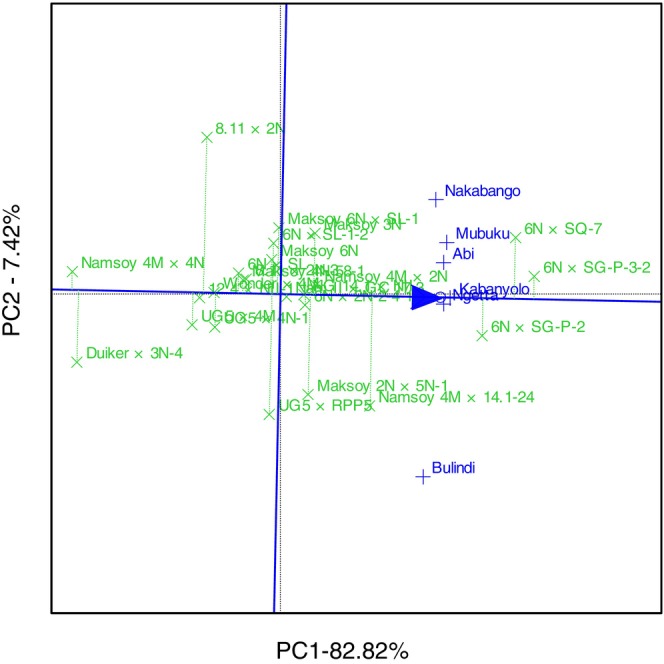
Performance and stability for hundred seed weight of 24 soybean genotypes tested across six locations for five cropping seasons in Uganda.

**FIGURE 5:**
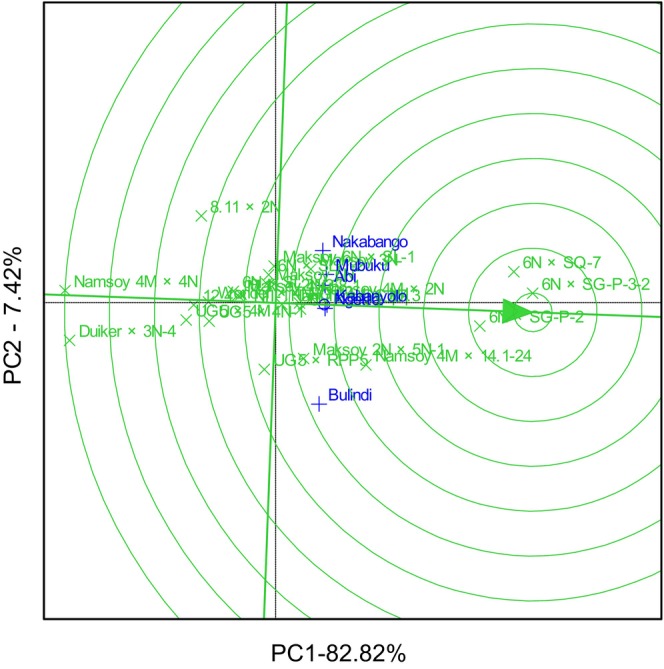
Ideal genotype analysis for hundred seed weight of 24 soybean genotypes tested across six locations for five cropping seasons in Uganda.

**FIGURE 6:**
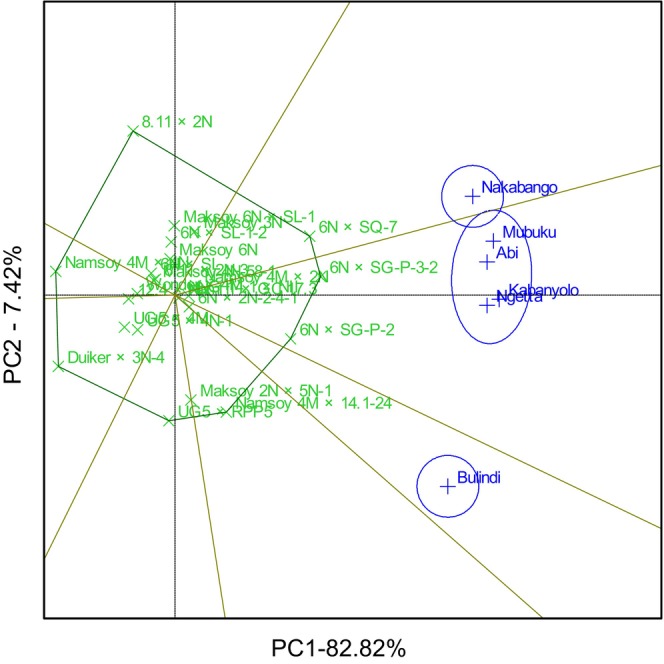
Mega environment analysis for hundred seed weight of 24 soybean genotypes tested across six locations for five cropping seasons in Uganda.

GGE biplot analysis explained 90.24% of the total GEI variation for hundred seed weight. The GGE biplot identified 6N × SG‐P‐3‐2, 6N × SG‐P‐2 and 6N × SQ‐7 with high and moderately stable genotypes for hundred seed weight. On the other hand, the GGE biplot identified Namsoy 4M × 4N with the lowest and moderate stability for hundred seed weight and the most unstable genotypes included 8.11 × 2N and UG5 × RPP5 (Figure 4). GGE biplot identified 6N × SG‐P‐3‐2 as the most ideal genotype, followed by 6N × SG‐P‐2 and 6N × SQ‐7. On the other hand, Namsoy 4M × 4N was the least ideal genotype compared to all the other genotypes (Figure 5). GGE biplot grouped the six locations into three mega‐environments. The first mega‐environment included Abi, Ngetta, Mubuku, Kabanyolo and 6N × SG‐P‐3‐2 being the vertex cultivar. The second mega‐environment included Bulindi, and the last mega‐environment included Nakabango and 6N × SQ‐7 as the vertex cultivar (Figure 6).

### 3.3 Grain Yield (kg ha^−1^)

#### 3.3.1 Performance of Genotypes Across Locations

The genotype, location, season, genotype × location, and genotype × location × season interactions for grain yield were highly significant (*p* ≤ 0.001) as presented in Table 6. Genotypes 6N × SG‐P‐3‐2, 6N × SG‐P‐2 and 6N × SL‐1‐2 had the highest mean grain yield across the six locations, with mean yields of 2085, 2053 and 1772 kg ha^−1^, respectively (Table 7). Several genotypes had higher yields than the check varieties Maksoy 3N and Maksoy 6N (Table 7). Bulindi recorded the highest mean grain yield of 2078 kg ha^−1^, followed by Mubuku (1998 kg ha^−1^), while Abi recorded the lowest mean grain yield of 1175 kg ha^−1^ (Table 7).

**TABLE 6:** Analysis of variance of grain yield (kg ha^−1^) of 24 soybean genotypes tested across six locations for five cropping seasons in Uganda.

SOV	df	Sum sq	Mean sq	*F* value	Pr (> *F*)
Genotype	23	29,780,733	1,294,814	17.923	< 2e−16
Location	6	1.03e+08	17,238,454	238.618	< 2e−16
Rep	2	1,378,905	689,452	9.544	7.74e−05
Season	4	1.41e+08	35,186,550	487.058	< 2e−16
Genotype × Location	134	19,555,778	145,939	2.02	9.03e−10
Genotype × Season	92	7,103,930	77,217	1.069	0.315
Location × Season	19	1.57e+08	8,265,071	114.407	< 2e−16
Genotype × Location × Season	413	44,323,781	107,322	1.486	2.19e−07
Residuals	1178	85,102,259	72,243		

**TABLE 7:** Grain yield (kg ha^−1^) of 24 soybean genotypes tested across six locations for five cropping seasons in Uganda.

Genotype	Abi	Bulindi	Kabanyolo	Mubuku	Nakabango	Ngetta	Mean
6N × SG‐P‐3‐2	1476	2279	2438	2727	2279	1496	2085
6N × SG‐P‐2	1353	1857	2601	2637	2226	1639	2053
6N × SL‐1‐2	1424	1911	1896	2342	1694	1474	1772
Maksoy 6N	1333	2111	1866	2265	1594	1355	1708
6N × SL	1258	2291	1802	2125	1622	1418	1688
Namsoy 4M × 14.1‐24	1192	2163	2038	2148	1541	1316	1672
6N × SQ‐7	1218	1517	2432	1920	1895	1026	1656
Maksoy 6N × SL‐1	1309	2218	1575	2071	1741	1334	1654
NG 14.1 × NII	1308	2119	1856	2132	1573	1165	1637
Wonder × 4M	1276	2239	1881	1905	1660	1248	1633
Maksoy 4N‐58‐1	1299	2170	1901	1809	1520	1420	1622
Nam II × GC 17.3	1079	2278	2036	2083	1517	1121	1604
Maksoy 3N	1168	2068	1947	2128	1416	1231	1602
8.11 × 2N‐3	1171	1924	1963	1869	1526	1244	1568
UG5 × 4N‐1	1223	2628	1725	1691	1390	1234	1532
8.11 × 2N	1166	1950	1773	1934	1397	1127	1504
12.4 × NII‐1	1049	2134	1728	2023	1406	1020	1488
UG5 × 4M	1035	2064	1719	2043	1340	950	1455
UG5 × RPP5	1013	2431	1714	1731	1236	994	1407
Namsoy 4M × 4N	862	2063	1662	1698	1339	1158	1387
Namsoy 4M × 2N	1124	1678	1809	1617	1326	1009	1384
6N × 2N‐2‐4‐1	917	1766	1544	1849	1316	1138	1377
Duiker × 3N‐4	952	2245	1685	1573	1265	1085	1369
Maksoy 2N × 5N‐1	985	1773	1607	1622	1173	1105	1323
Mean	1175	2078	1883	1998	1541	1221	1591

#### 3.3.2 Stability Using GGE Biplot Analysis

**FIGURE 7:**
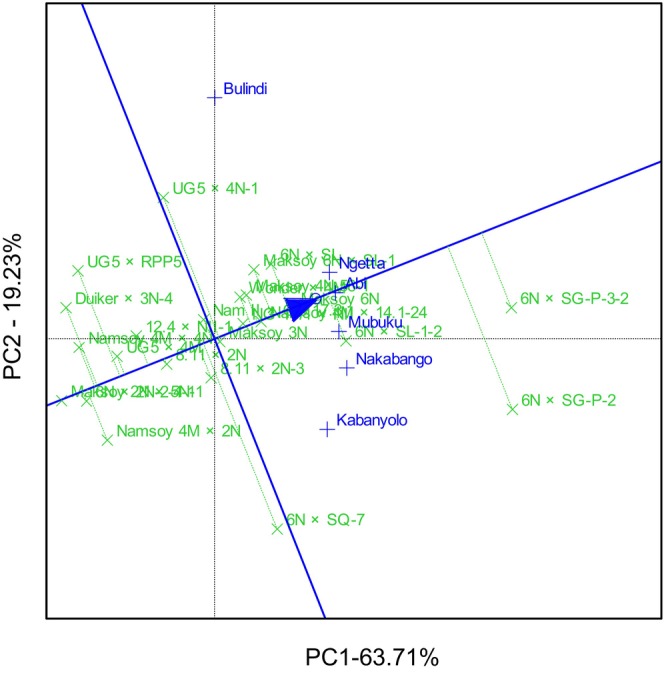
The mean performance view versus stability of the genotype for grain yield of 24 soybean genotypes tested across six locations for five cropping seasons in Uganda.

**FIGURE 8:**
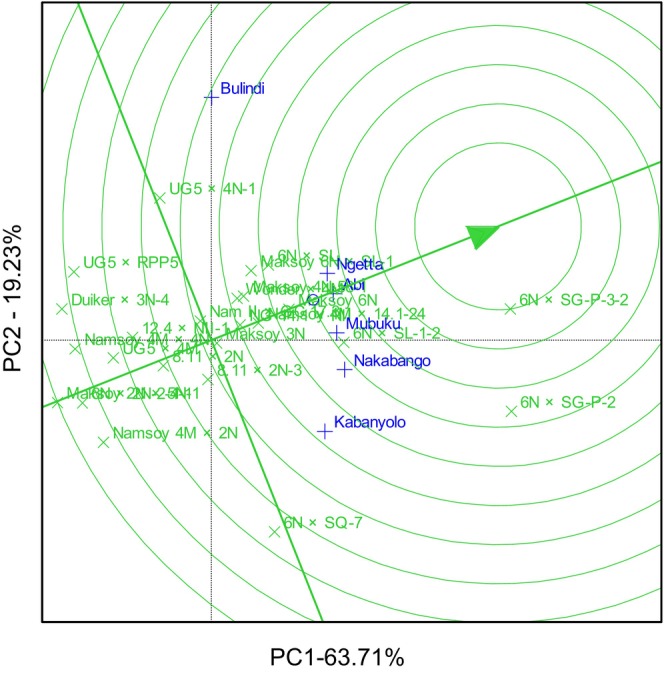
Ideal genotype analysis for grain yield of 24 soybean genotypes tested across six locations for five cropping seasons in Uganda.

**FIGURE 9:**
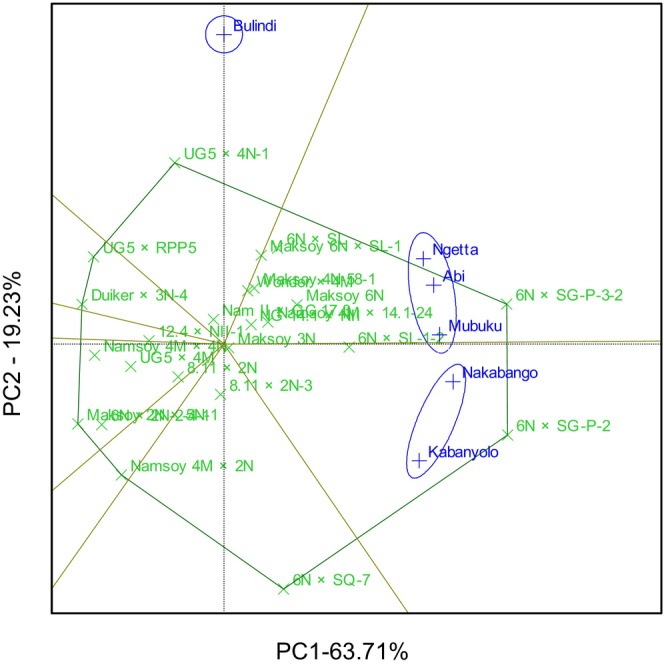
The which won where view of the genotypes for grain yield of 24 soybean genotypes tested across six locations for five cropping seasons in Uganda.

GGE biplot analysis explained 82.94% of the total GEI variation for grain yield and identified 6N × SG‐P‐3‐2 as the highest‐yielding genotype, followed by 6N × SG‐P‐2 (Figure 7). The GGE biplot also illustrated that the lowest‐yielding genotypes were Maksoy 2N × 5N‐1 and 6N × 2N‐2‐4‐1 (Figure 7). GGE biplot identified 6N × SG‐P‐3‐2 as the most ideal genotype, implying it had high and stable grain in all six locations (Figure 8). On the other hand, Maksoy 2N × 5N‐1 was the least ideal genotype (Figure 8). GGE biplot grouped the six locations into three mega‐environments for grain yield. The first mega‐environment included Abi, Ngetta, and Mubuku, with 6N × SG‐P‐3‐2 being the vertex cultivar. The second mega‐environment included Bulindi and UG5 × 4N‐1 as the vertex cultivar, and the last mega‐environment included Nakabango and Kabanyolo, with 6N × SG‐P‐2 as the vertex cultivar (Figure 9).

## 4 Discussion

### 4.1 Soybean Rust Resistance Scores

Generally, low soybean rust resistance scores were observed across the test lines in this study, aligning with previous findings from Uganda (Tukamuhabwa et al. 2012, 2019). This trend is attributed to the fact that resistance to soybean rust has been a major selection criterion in the Ugandan soybean breeding program (Tukamuhabwa and Oloka 2016; Tukamuhabwa et al. 2019). The low disease scores among advanced breeding lines highlight the effectiveness of ongoing selection efforts aimed at improving resistance to soybean rust disease. In addition, several test lines demonstrated significantly lower soybean rust scores compared to the check varieties Maksoy 3N and Maksoy 6N. Notably, four of the top five most resistant genotypes in this study had Maksoy 6N as a parent. This suggests that Maksoy 6N may possess strong general combining ability (GCA) for soybean rust resistance, contributing favorable alleles to its progeny. These results highlight Maksoy 6N as a valuable parental line in breeding programs aimed at improving resistance to soybean rust and emphasize the importance of evaluating GCA when selecting parents for soybean rust disease resistance breeding. Although Maksoy 3N was previously reported to exhibit partial resistance to soybean rust (Murithi et al. 2016), it recorded a relatively high disease score of 2.6 in the current study. This suggests that Maksoy 3N may be losing its effectiveness against the pathogen. The observed increase in disease severity on previously resistant varieties implies the presence of virulent pathogen populations in Uganda, potentially overcoming existing resistance genes. Therefore, routine screening of germplasm is essential to track pathogen evolution and ensure the durability of resistance sources.

The study further revealed a highly significant genotype‐by‐environment interaction (GEI) for soybean rust resistance scores, indicating that environmental factors play a substantial role in the expression of resistance across different locations (Murithi et al. 2021). This interaction reflects the variability in climatic conditions, particularly humidity and temperature, which are critical drivers of soybean rust disease development. Soybean rust disease develops rapidly at moderate to warm temperatures (15°C–30°C), and its severity increases under high relative humidity levels exceeding 75%–80%, particularly when leaves remain wet for extended periods (Malvick 2018). For instance, Kabanyolo and Nakabango recorded high soybean rust scores. These two locations are located in the Lake Victoria Crescent agro‐ecological zone, characterized by high humidity and warm temperatures—conditions that are highly conducive to the proliferation of *P. pachyrhizi*, the fungal pathogen responsible for soybean rust (Sweets et al. 2008). Similarly, Mubuku exhibited elevated disease scores, likely due to flood irrigation practices, which increase ambient humidity and create a microclimate favorable for disease development. These findings underscore the importance of evaluating genotypes under diverse environmental conditions to effectively identify stable resistance sources and inform breeding strategies tailored to specific agroecologies.

The GGE biplot analysis showed that the six locations were grouped into two mega‐environments for soybean rust scores. Nakabango, Mubuku and Kabanyolo were grouped in one mega environment because they shared similar climatic conditions due to the high humidity. Nakabango and Kabanyolo are characterized by high levels of humidity because they both belong to the Lake Victoria crescent agroecological zone, which is characterized by high relative humidity that favors the growth and development of *P. pachyrhizi*, the pathogen that causes soybean rust disease. Mubuku, on the other hand, was grouped with those two locations because of the high level of humidity due to the use of flood irrigation in this specific location. Additionally, two locations, Abi and Ngetta were grouped in this mega environment. These two locations are characterized by low relative humidity and high temperatures. Therefore, they were grouped in this mega environment because of the high temperatures that favor the growth and development of *P. pachyrhizi*, the pathogen that causes soybean rust disease. The best cultivar for soybean rust resistance in this mega environment is 6N × SQ‐7 because it is the vertex cultivar.

The second mega‐environment had only one location, Bulindi, with 6 N × SG‐P‐3‐2 as the vertex cultivar. It was not surprising that Bulindi was grouped in a separate mega environment. This location is located in the Midwest agroecological zone, which is characterized by completely different climatic conditions, characterized by high humidity and low temperatures. Observation from this study implies that high relative humidity and high temperatures strongly favor soybean rust growth and development, as reported in previous studies (Maphosa 2013; Tukamuhabwa and Maphosa 2011; Tukamuhabwa et al. 2012).

The GGE biplot analysis revealed that the six test locations clustered into two distinct mega‐environments based on soybean rust disease scores. Nakabango, Mubuku, and Kabanyolo formed one mega‐environment, likely due to their shared high‐humidity conditions. Nakabango and Kabanyolo are situated within the Lake Victoria Crescent agro‐ecological zone, which is characterized by consistently high relative humidity, creating ideal conditions for the growth and development of *P. pachyrhizi*, the causal agent of soybean rust. Mubuku, although located outside this zone, was grouped with these locations due to the increased humidity resulting from flood irrigation practices. Interestingly, Abi and Ngetta were also grouped in this mega‐environment. Despite being characterized by low relative humidity and high temperatures, their inclusion suggests that elevated temperatures may significantly promote the development of soybean rust, even in less humid environments. The vertex genotype for this mega‐environment was 6N × SQ‐7, indicating it had the highest performance and stability for rust resistance under these conditions. The second mega‐environment consisted solely of Bulindi, with 6N × SG‐P‐3‐2 identified as the vertex genotype. Bulindi, located in the Midwest agro‐ecological zone, experiences distinct climatic conditions, notably high humidity coupled with lower temperatures, which likely influenced its unique grouping. These findings reinforce the conclusion that soybean rust development is favored by both high humidity and high temperature, aligning with previous research (Maphosa 2013; Tukamuhabwa and Maphosa 2011; Tukamuhabwa et al. 2012). The observed mega‐environment differentiation underscores the need for location‐specific breeding and deployment strategies to enhance soybean rust resistance.

### 4.2 Hundred Seed Weight (HSW)

Additionally, several test lines showed significantly higher hundred‐seed weight than the check varieties Maksoy 3N and Maksoy 6N, suggesting effective progress in selection for seed size in Uganda. Currently, the industries in Uganda and across the East African region prefer soybean varieties with a large hundred‐seed size. Additionally, the current study further observed a small variation between the highest hundred weight (20 g) and the lowest (14 g). This observation is similar to previous studies that reported similar ranges (Abebe et al. 2024; Hina et al. 2020; Kumar et al. 2023; Ray et al. 2022).

The current study revealed significant differences in hundred‐seed weight among genotypes, locations, seasons, and genotype × location interactions. This aligns with previous findings that report hundred‐seed weight as a quantitative trait governed by multiple genes and strongly influenced by environmental conditions (Hina et al. 2020; Kumar et al. 2023). The observed variation in hundred‐seed weight across different Ugandan locations reinforces the substantial environmental influence on this trait. Notably, several test lines exhibited significantly higher hundred‐seed weights than the check varieties Maksoy 3N and Maksoy 6N, suggesting significant genetic gain and progress in selection for larger seed size within the Ugandan breeding program. This is particularly relevant given that soybean grain processors in Uganda and the broader East African region prefer varieties with larger hundred‐seed weights due to their processing efficiency.

Furthermore, the range in hundred‐seed weight among the evaluated genotypes was relatively narrow, varying between 14 and 20 g. This limited variation may be attributed to the shared genetic background of the test genotypes, which likely constrains the expression of broader phenotypic diversity for this trait. This limited variability is consistent with prior studies (Abebe et al. 2024; Hina et al. 2020; Kumar et al. 2023; Ray et al. 2022). The narrow range observed may indicate limited genetic diversity for seed size within the current breeding pool. This highlights the need to introduce novel germplasm with broader variability to avoid genetic stagnation and meet diverse market demands. In addition, genotypes demonstrating stable and superior seed weight across environments could serve as elite parents in crossing programs, ultimately supporting the development of high‐yielding, market‐preferred soybean cultivars.

In the current study, the GGE biplot identified 6N × SG‐P‐3‐2 as the most ideal genotype. This implies that this genotype had both high and stable hundred‐seed weight in the different locations and seasons.

The GGE biplot analysis in this study explained 90.24% of the total genotype × environment interaction (GEI) variation for hundred‐seed weight, indicating a strong model fit and reliability of the graphical interpretation for identifying high‐performing and stable genotypes. This high explanatory power suggests that the genotypic and GEI components captured by the first two principal components were sufficient to account for the variation in hundred‐seed weight across locations and seasons. Among the tested genotypes, 6N × SG‐P‐3‐2, 6N × SG‐P‐2, and 6N × SQ‐7 were identified as genotypes with both high hundred‐seed weight and moderate to high stability across environments. These lines represent promising candidates for further advancement and possible varietal release, given their consistent performance and adaptability. Notably, 6N × SG‐P‐3‐2 emerged as the most ideal genotype, possessing both superior yield and stability, making it particularly valuable for environments where consistency in seed size is critical for marketability and processing. Genotypes such as 8.11 × 2N and UG5 × RPP5 were classified as the most unstable, suggesting a strong sensitivity to environmental variation and limited suitability for broad adaptation. These findings underscore the importance of evaluating both performance and stability when selecting genotypes for release or as parental lines in breeding programs.

The GGE biplot further grouped the six testing locations into three distinct mega‐environments, revealing significant spatial variation in genotype performance. The first mega‐environment included Abi, Ngetta, Mubuku, and Kabanyolo, with 6N × SG‐P‐3‐2 serving as the vertex genotype, indicating its superior performance in these locations. The second mega‐environment was represented solely by Bulindi, suggesting unique environmental conditions that differentiate it from other sites. The third mega‐environment comprised Nakabango, where 6N × SQ‐7 was the vertex genotype. These groupings emphasize the importance of targeted breeding and genotype deployment strategies based on specific environmental conditions to maximize genetic gain and stability. Overall, the identification of high‐performing and stable genotypes, along with the delineation of mega‐environments, provides critical insights for soybean improvement efforts.

### 4.3 Grain Yield (kg ha^−1^)

The present study evaluated soybean genotypes across diverse environmental conditions and provided valuable insights into the influence of genotype (G), environment (E), and their interaction (GEI) on grain yield performance. The observed variation in yield and stability among genotypes was largely attributable to these three factors, reinforcing the importance of identifying genotypes that are well‐adapted or specifically responsive to particular environments. The top‐performing genotypes 6N × SG‐P‐3‐2, 6N × SG‐P‐2, and 6N × SL‐1‐2 consistently outperformed the check varieties Maksoy 3N and Maksoy 6N, which are widely adopted by Ugandan farmers. This finding indicates substantial progress in breeding for improved grain yield and highlights the potential of these lines as future cultivars or parents in yield‐focused breeding programs.

The significant genotype × environment interaction observed in this study confirms that grain yield is a complex quantitative trait, influenced by multiple genes and highly sensitive to environmental variability. Key factors such as rainfall distribution, temperature, and soil fertility likely contributed to the differential performance observed across locations. For instance, Bulindi, located in the Midwest agroecological zone, benefits from stable and well‐distributed rainfall, creating favorable growing conditions. In contrast, Mubuku, where flood irrigation is practised, supports aggressive vegetative growth and higher yields due to consistent moisture availability. These findings are consistent with previous studies, which showed that irrigation or a reliable water supply significantly enhances yield performance in multi‐environment trials (Obua et al. 2021; Obua 2013). As climate change continues to disrupt rainfall patterns, these results highlight the increasing importance of incorporating water management strategies, such as irrigation, into soybean production systems. Doing so may be essential for sustaining and improving yields in Uganda's variable agro‐ecological zones.

The GGE biplot further identified genotype 6N × SG‐P‐3‐2 as the ideal genotype, as it was positioned closest to the origin of the concentric circles, indicating both high mean grain yield and superior stability across environments. This reinforces its potential as a broadly adapted and high‐performing cultivar under diverse Ugandan agroecologies. Additionally, the biplot analysis grouped the six test locations into three distinct mega‐environments, each associated with a vertex genotype representing the best performer within that cluster. For instance, 6N × SG‐P‐3‐2, which recorded the highest grain yields in Abi, Ngetta, and Mubuku, was identified as the vertex cultivar for that mega‐environment and may be specifically recommended for regions sharing similar environmental conditions. This targeted recommendation could improve yield potential by aligning genotype adaptation with local agro‐climatic conditions. The delineation of test locations into mega‐environments suggests increasing agro‐ecological stratification across Uganda, potentially driven by ongoing climatic shifts. Such segmentation provides a valuable framework for breeding programs to develop and deploy region‐specific soybean varieties, particularly in areas vulnerable to climate extremes such as prolonged droughts or rising temperatures. Tailoring varietal development to specific environmental challenges will be critical to ensuring resilient and sustainable soybean production in the face of climate variability.

The combined evaluation of soybean rust resistance, hundred‐seed weight (HSW), and grain yield in this study provides a comprehensive understanding of the performance and breeding potential of the tested genotypes. Genotypes such as 6N × SG‐P‐3‐2 and 6N × SG‐P‐2 emerged as particularly promising, exhibiting high grain yield, stable and desirable seed size, and moderate resistance to soybean rust. This is especially important as soybean rust can significantly reduce both yield and seed quality, threatening profitability and food security. Moreover, larger seed size, as reflected in higher HSW, aligns with market and processing preferences in Uganda and the broader East African region. The results emphasize the value of a multi‐trait selection approach in soybean breeding. Rather than selecting solely for high yield, integrating selection for disease resistance and seed size ensures that resulting cultivars are not only productive but also resilient and commercially viable. Multi‐trait selection helps breeders identify genotypes with balanced performance, reducing trade‐offs between agronomic and quality traits while increasing the likelihood of stable performance across diverse environments. The significant genotype × environment interactions observed for all three traits further underscore the need to select genotypes that can consistently express favorable trait combinations under variable climatic and ecological conditions. This integrative strategy is crucial for sustainable soybean improvement in Uganda, where climate variability and evolving pathogen pressures demand cultivars that can perform reliably across agro‐ecological zones while meeting market and production system needs.

## 5 Conclusion

This study successfully identified promising soybean genotypes with superior performance for soybean rust resistance, hundred‐seed weight (HSW) and grain yield under diverse agro‐ecological conditions in Uganda. Significant genotypic variation and genotype × environment interactions (GEI) were observed for all three traits, underscoring the importance of strategic selection. Notably, 6N × SG‐P‐3‐2, 6N × SG‐P‐2, and 6N × SQ‐7 emerged as top‐performing genotypes, exhibiting high and stable yields, desirable seed size, and moderate resistance to soybean rust. These genotypes were evaluated in an advanced yield trial, and findings from this study will support their progression into on‐farm trials and variety release.

The results demonstrate the value of a multi‐trait selection strategy, which balances agronomic performance, disease resistance, and seed quality to meet both production and market demands. This approach is critical for enhancing food security and farmer profitability, especially under conditions of climate variability and evolving disease pressure. Furthermore, the identification of three mega‐environments provides a framework for environment‐specific genotype deployment, improving breeding efficiency and varietal targeting across Uganda's diverse agro‐ecological zones.

However, the study had some limitations. First, it did not include molecular characterization of rust resistance genes, which limits the understanding of the underlying genetic mechanisms. Second, no correlation analysis was conducted to determine the relationship among soybean rust resistance, grain yield, and hundred‐seed weight, which could have provided further insights into potential trade‐offs or synergies among these traits. Future research should integrate molecular tools and trait relationship analyses like correlations and multi‐trait selection, and pathways to enhance selection precision and the possibility of gene pyramiding for these traits in one genotype. In addition, irrigation and water management strategies, particularly in drought‐prone areas, are recommended to ensure yield stability across seasons. The adoption and release of these superior genotypes hold strong potential to contribute to sustainable soybean production, improved farmer livelihoods, and enhanced agro‐industrial competitiveness in Uganda and the wider East African region.

## Funding

The research was funded by Makerere University Centre for Soybean Improvement and Development (MAKCSID) and International Institute of Tropical Agriculture (IITA).

## Conflicts of Interest

The authors declare no conflicts of interest.

## Supporting information


**Table S1:** pei370125‐sup‐0001‐TableS1.xlsx.

## Data Availability

Data is available in the Supporting Information.
